# Successful Treatment of Postprandial Hyperinsulinemic Hypoglycemia After Billroth-II Gastrojejunostomy Using Octreotide

**DOI:** 10.1210/jcemcr/luad150

**Published:** 2023-12-01

**Authors:** Masashi Hasebe, Megumi Aizawa-Abe, Kimitaka Shibue, Akihiro Hamasaki

**Affiliations:** Department of Diabetes and Endocrinology, Medical Research Institute Kitano Hospital, PIIF Tazuke-Kofukai, Kita-ku, Osaka 530-8480, Japan; Department of Diabetes and Endocrinology, Medical Research Institute Kitano Hospital, PIIF Tazuke-Kofukai, Kita-ku, Osaka 530-8480, Japan; Division of Diabetes and Endocrinology, Osaka Saiseikai-Noe Hospital, Osaka 536-0001, Japan; Department of Diabetes and Endocrinology, Medical Research Institute Kitano Hospital, PIIF Tazuke-Kofukai, Kita-ku, Osaka 530-8480, Japan; Department of Diabetes and Endocrinology, Medical Research Institute Kitano Hospital, PIIF Tazuke-Kofukai, Kita-ku, Osaka 530-8480, Japan

**Keywords:** gastrointestinal surgery, hyperinsulinemic hypoglycemia, GLP-1, GIP, octreotide

## Abstract

Postprandial hyperinsulinemic hypoglycemia, although rare, is a well-documented complication that can manifest after upper gastrointestinal surgery. Despite its potential for severe morbidity, the underlying pathogenesis and optimal treatment strategies for this condition remain insufficiently understood. This report presents a compelling case of postprandial hypoglycemia following Billroth-II gastrojejunostomy, characterized by a marked increase in postprandial insulin levels, accompanied by the exaggerated response of incretin hormones. The incretin effect in this patient was found to be exceptionally high, measuring at approximately 90%. While nutritional interventions proved ineffective in alleviating the patient's symptoms, the administration of octreotide significantly attenuated the exaggerated postprandial insulin and incretin response, substantially ameliorating both the symptoms and postprandial hypoglycemia. Monthly subcutaneous injections of long-acting repeatable octreotide were initiated, resulting in the complete resolution of symptomatic postprandial hypoglycemia. Although the patient developed acalculous cholecystitis and gallstone cholangitis 2 years after commencing octreotide therapy, she has remained free from symptomatic postprandial hypoglycemia for more than 4 years. Our case underscores the efficacy of somatostatin analogs in the management of postprandial hyperinsulinemia after gastrointestinal surgery, shedding light on the potential involvement of incretin hormones in the pathophysiology of this condition.

## Introduction

Postprandial hyperinsulinemic hypoglycemia is a rare yet well-documented complication that can arise subsequent to upper gastrointestinal surgery (1). Despite its potential for severe morbidity, including neuroglycopenia, the underlying pathogenesis and optimal treatment strategies for this condition remain poorly elucidated.

This report presents a compelling case of postprandial hypoglycemia following Billroth-II gastrojejunostomy. This case is characterized by a pronounced increase in postprandial insulin levels, accompanied by the hypersecretion of incretin hormones, namely glucagon-like peptide-1 (GLP-1) and glucose-dependent insulinotropic polypeptide (GIP). Notably, the administration of octreotide significantly attenuated the exaggerated post-meal insulin and incretin response, substantially ameliorating the patient's symptoms and postprandial hypoglycemia. Our case underscores the efficacy of somatostatin analogs in the management of postprandial hyperinsulinemia after gastrointestinal surgery, shedding light on the potential involvement of incretin hormones in the pathophysiology of this condition.

## Case Presentation

An 84-year-old Japanese woman (height: 146.5 cm, weight: 60.2 kg, body mass index [BMI]: 28.0 kg/m^2^) was referred to our hospital with repeated episodes of postprandial hypoglycemia. Her medical history included a Billroth-II gastrojejunostomy procedure for gastric cancer and a small bowel resection of approximately 1 meter due to postoperative bowel obstruction, performed 35 years prior to her current presentation. After these surgeries, she began experiencing symptoms of postprandial hypoglycemia, such as cold sweats and palpitations, which have progressively worsened, particularly over the past 5 years. Notably, she did not experience hypoglycemic symptoms during fasting periods. While she was prescribed antihypertensive medication for hypertension, no medications were identified as potential inducers of hypoglycemia. Abdominal computed tomography scans revealed no apparent pancreatic tumors.

Given the patient's history of previous gastrojejunostomy and the recurrent hypoglycemic symptoms occurring after meals rather than during fasting, we considered the possibility of postprandial hyperinsulinemic hypoglycemia associated with gastrectomy. Consequently, we initiated a comprehensive investigation into the pathophysiology of glucose metabolism following nutrient intake.

## Diagnostic Assessment

**Figure 1.**
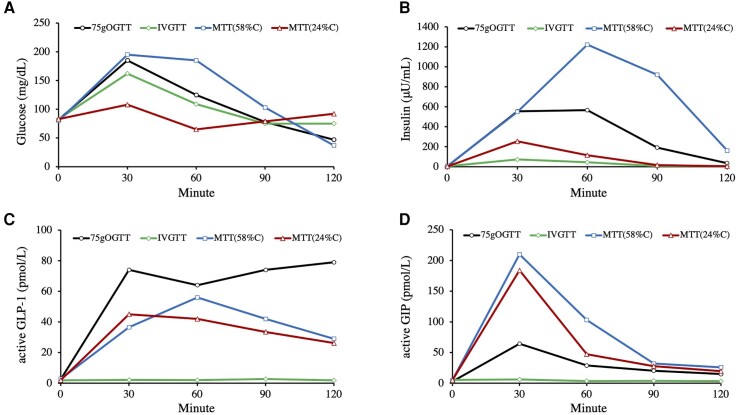
(A) Plasma glucose (mg/dL), (B) serum insulin (μU/mL), (C) plasma active glucagon-like peptide-1 (GLP-1) (pmol/L), and (D) plasma active glucose-dependent insulinotropic polypeptide (GIP) (pmol/L) during the 75-g oral glucose tolerance test (OGTT), 2 meal tolerance tests (MTTs) with varying carbohydrate compositions (24% and 58%), and the isoglycemic intravenous glucose infusion test (IVGTT). For the 2 MTTs, one meal consisted of 460 kcal with a composition of 24% carbohydrates, 19% protein, and 57% fat (24%C), while the other isocaloric meal consisted of 58% carbohydrates, 8% protein, and 34% fat (58%C). The IVGTT was designed to mimic the glucose fluctuations observed during the 75-g OGTT.

After an overnight fast, we conducted a 75-g oral glucose tolerance test (OGTT) and observed a substantial increase in serum insulin levels shortly after glucose ingestion, followed by hypoglycemia manifesting 120 minutes after the administration of glucose (reaching 47 mg/dL [2.6 mmol/L]) (Fig. 1A and 1B). Subsequently, we performed 2 meal tolerance tests (MTTs). The first test meal contained 460 kcal, comprising 24% carbohydrates, 19% protein, and 57% fat, while the second test meal was isocaloric, consisting of 58% carbohydrates, 8% protein, and 34% fat. The first MTT revealed low blood glucose after 60 minutes of ingestion (65 mg/dL [3.6 mmol/L]), while the second meal test showed even lower blood glucose levels after 120 minutes of ingestion (37 mg/dL [2.1 mmol/L]) (Fig. 1A). It is noteworthy that the latter test exhibited more pronounced insulin secretion (peak value: 1222 μU/mL [8768 pmol/L] at 60 minutes) and subsequent hypoglycemia (Fig. 1B). Remarkably, all 3 tests indicated an excessive secretion of active GLP-1 and GIP following glucose or meal intake (peak values: active GLP-1 45-79 pmol/L and active GIP 64-210 pmol/L) (Fig. 1C and 1D). These peak levels, particularly GLP-1, were notably higher than those seen in healthy Japanese individuals (peak values in MTT: active GLP-1 0.7 ± 0.2 pmol/L and active GIP 70.3 ± 10.2 pmol/L) (2). Furthermore, we carried out an isoglycemic intravenous glucose infusion test (IVGTT) designed to replicate the glucose fluctuations observed in the 75-g OGTT. During this test, as expected, the secretion of insulin, GLP-1, and GIP was significantly lower than the levels observed after oral glucose intake (Fig. 1B-1D). To assess the incretin effect, we employed the following formula: (AUC for OGTT − AUC for IVGTT)/AUC for IVGTT. Here, AUC represents the incremental (above baseline) area under the curves for serum insulin during OGTT or IVGTT. This calculation indicated a markedly heightened incretin effect in this patient (∼90%), underscoring a unique and pronounced physiological response. The hypersecretion of insulin following oral glucose or meal consumption led to a diagnosis of hypoglycemia stemming from the previous gastrojejunostomy, and we postulated the potential involvement of an exaggerated incretin effect.

## Treatment

**Figure 2.**
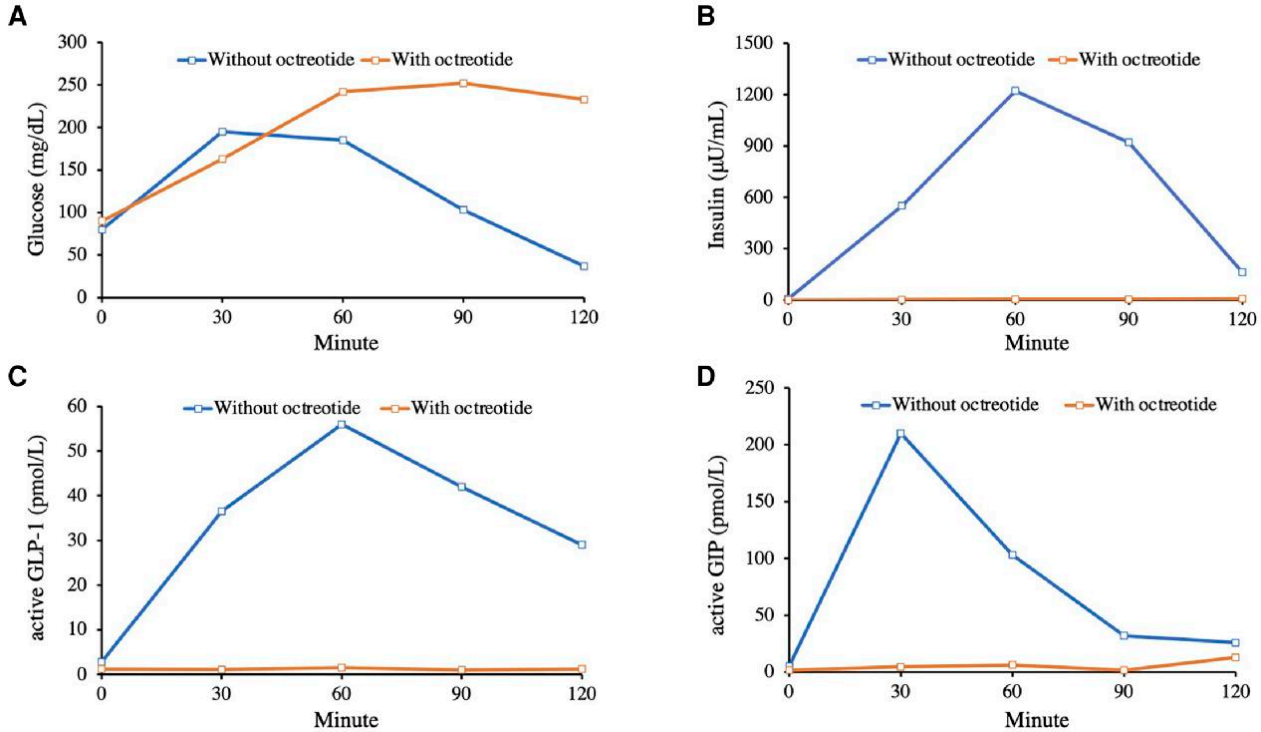
(A) Plasma glucose (mg/dL), (B) serum insulin (μU/mL), (C) plasma active glucagon-like peptide-1 (GLP-1) (pmol/L), and (D) plasma active glucose-dependent insulinotropic polypeptide (GIP) (pmol/L) during the meal tolerance test with an energy content of 460 kcal and carbohydrate content of 58%, without or with a prior subcutaneous injection of 25 μg octreotide.

Despite implementing nutritional interventions, such as frequent consumption of small meals high in protein and fiber and avoiding high glycemic index carbohydrates, the patient's postprandial hypoglycemic symptoms showed no improvement. After an overnight fast, we conducted another MTT with an energy content of 460 kcal and carbohydrate content of 58% 1 hour after a subcutaneous injection of 25 µg octreotide, a somatostatin analog. This intervention significantly reduced the excessive secretion of insulin, active GLP-1, and active GIP (peak values: insulin 6.8 μU/mL [48.8 pmol/L], active GLP-1 1.5 pmol/L, and active GIP 12.9 pmol/L), resulting in stable blood glucose levels after the meal (Fig. 2). Subsequently, upon initiating a monthly subcutaneous injection of long-acting repeatable octreotide at a dose of 20 mg, the patient's postprandial hypoglycemic symptoms wholly resolved, leading to a substantial enhancement in the patient's overall quality of life.

## Outcome and Follow-Up

After initiating the monthly subcutaneous injections of long-acting repeatable octreotide at a dose of 20 mg, the patient experienced no adverse events and maintained a high quality of life. Furthermore, as the need for frequent food intake to prevent hypoglycemia became obsolete, body weight decreased and subsequently stabilized within the standard range, resulting in a BMI of approximately 20 kg/m^2^. However, it is worth noting that the patient developed acalculous cholecystitis and gallstone cholangitis 2 years after starting octreotide treatment, both of which were effectively managed with antimicrobial agents and endoscopic biliary drainage. Suspecting that these events may have been related to octreotide, and taking into account the patient's loss of body weight and concerned sarcopenia, we cautiously reduced the dose of long-acting repeatable octreotide to 6 mg monthly, aiming to mitigate the risk of adverse events. Eventually, more than 4 years of treatment have transpired without any symptomatic postprandial hypoglycemia, and no apparent adverse events were observed after the dose reduction of octreotide. Throughout the treatment course, glycated hemoglobin levels consistently remained within the normative or prediabetic range (5.0%-6.0% [31-42 mmol/mol]).

## Discussion

We have described a case involving postprandial hyperinsulinemic hypoglycemia in a patient who underwent a Billroth-II gastrojejunostomy. Consuming oral glucose or meals triggered an excessive insulin release, along with an elevated release of incretin hormones and an amplified incretin effect. However, administering octreotide effectively suppressed the secretion of both insulin and incretin hormones, leading to an improvement in symptomatic postprandial hypoglycemia. The current case reiterates the role of hypersecretion of incretin hormones in postprandial hyperinsulinemic hypoglycemia following upper gastrointestinal surgery and highlights the effectiveness of somatostatin analogs in its treatment.

Postprandial hypoglycemia can be a potential complication following upper gastrointestinal surgery, including bariatric procedures (1, 3). For some patients, it may manifest severely and significantly impact their quality of life, leading to substantial morbidity. Although the precise pathophysiology underlying postoperative hypoglycemia remains elusive, several explanations have been proposed. One theory posits that it relates to the oversecretion of incretin hormones in response to rapid nutrient delivery to the small intestine, the primary site of incretin production, as observed in the present case. This surge in incretin hormones is thought to trigger excessive insulin secretion in a dose-dependent manner, thus precipitating postprandial hypoglycemia (3, 4). In addition to their insulin-stimulating effects, incretin hormones also function as trophic factors for pancreatic β cells, potentially causing β cell hypertrophy. Consequently, it is conceivable that the development of pancreatic β cell hypertrophy, referred to as nesidioblastosis, induced by incretin hormones, may also contribute to postoperative hypoglycemia (5). Although we cannot definitively establish the role of nesidioblastosis in the current case due to the patient not undergoing pancreatectomy, it is plausible that the gradual hypertrophy of pancreatic β cells stimulated by incretin hormones may be linked to the long-term deterioration of her symptomatic hypoglycemia.

The cornerstone of treatment for postprandial hypoglycemia after gastrointestinal surgery lies in nutritional therapy, encompassing frequent consumption of small meals rich in protein and fiber while low in fat and high glycemic index carbohydrates (1). Nevertheless, such dietary modifications are challenging to sustain and often prove ineffective. If nutritional therapy alone fails to prevent hypoglycemia, pharmacological intervention becomes necessary. However, medical treatments, including α-glucosidase inhibitors, diazoxides, and calcium channel blockers, frequently yield unsatisfactory results, and a definitive treatment algorithm has yet to be established (1). Several case reports have documented the efficacy of somatostatin analogs in managing postprandial hyperinsulinemic hypoglycemia following upper gastrointestinal surgery (6). Somatostatin receptors (SSTRs) comprise 5 isoforms (SSTR1-SSTR5). The isoforms SSTR1, SSTR2, and SSTR5 serve as mediators of insulin secretion inhibition within human pancreatic β cells (7), while SSTR5 is reported to be expressed in rodent L and K cells (8, 9). In patients experiencing postprandial hypoglycemia after gastrointestinal surgery, octreotide, known for its strong affinity for SSTR2 and SSTR5 receptors, has shown the ability not only to curtail insulin responses but also to dampen the secretion of GLP-1 and GIP, as seen in our case (6). In light of a study demonstrating the effectiveness of GLP-1 receptor antagonists in rectifying postprandial hypoglycemia among patients who have undergone gastric bypass surgery (10, 11), it is conceivable that the exaggerated release of incretin hormones contributes significantly to postprandial hypoglycemia, and therefore, the suppression of these hormones through octreotide administration played a pivotal role in alleviating hypoglycemia in the current case. In the future, avexitide, a GLP-1 receptor antagonist, might also emerge as a promising treatment option for this condition, given its efficacy in a clinical trial (11).

The use of somatostatin analogs has been hindered due to potential side effects, including diarrhea, steatorrhea, cholelithiasis, and QT prolongation. In our present case, while the acalculous cholecystitis and gallstone cholangitis developed 2 years after the commencement of octreotide therapy, the patient safely continued octreotide therapy for more than 4 years without experiencing any other adverse effects. In line with our observations, a case report has documented the successful use of somatostatin analogs in the management of postprandial hypoglycemia following gastric bypass surgery for a period exceeding 4 years (6). These promising results indicate that somatostatin analogs offer a potential long-term therapeutic solution for postprandial hypoglycemia following upper gastrointestinal surgery. Nevertheless, it is also crucial to note that in our current case, the patient exhibited postprandial hyperglycemia during the MTT following octreotide administration (2-hour glucose 233 mg/dL [12.9 mmol/L]) (Fig. 2A). Fortunately, in our case, the patient remained euglycemic or prediabetic during the treatment course. Nevertheless, it is essential to closely monitor blood glucose levels.

We describe a case of postprandial hyperinsulinemic hypoglycemia with hypersecretion of incretin hormones following a Billroth-II gastrojejunostomy. Octreotide effectively alleviated postprandial hypoglycemia, showcasing the potential of somatostatin analogs in treating hormonal imbalances after gastrointestinal surgery. Our case emphasizes the effectiveness of these analogs in managing postprandial hypoglycemia following such procedures, while also stressing the importance of vigilant monitoring for potential side effects.

## Learning Points

- After undergoing upper gastrointestinal surgery, patients may face a substantial decline in their quality of life due to symptomatic postprandial hypoglycemia, which can persist long after the surgical procedure.
- Postprandial hypoglycemia following upper gastrointestinal surgery results from meal-induced insulin hypersecretion, with the crucial contribution of an exaggerated response in incretin hormones.
- Despite the limited success of nutritional therapy and various medications in managing postprandial hyperinsulinemic hypoglycemia, somatostatin analogs have shown their effectiveness by potently inhibiting incretin and insulin secretion.
- Given the favorable long-term efficacy of somatostatin analogs, they are a promising treatment for postprandial hypoglycemia after upper gastrointestinal surgery; however, careful monitoring for potential side effects is essential.

## Contributors

All authors made individual contributions to authorship. M.A-A. and A.H. were involved in the diagnosis and management of this patient. M.H. and K.S. were involved in writing the first draft of the manuscript, which was critically revised by M.A-A. and A.H. All authors read and approved the final draft.

## Data Availability

Data sharing is not applicable to this article as no data sets were generated or analyzed during the current study.

## Abbreviations

BMI: body mass index
GIP: glucose-dependent insulinotropic polypeptide
GLP-1: glucagon-like peptide-1
IVGTT: intravenous glucose tolerance test
MTT: meal tolerance test
OGTT: oral glucose tolerance test
SSTR: somatostatin receptor
